# Effectiveness of a Sodium-Reduction Smartphone App and Reduced-Sodium Salt to Lower Sodium Intake in Adults With Hypertension: Findings From the Salt Alternatives Randomized Controlled Trial

**DOI:** 10.2196/43675

**Published:** 2023-03-09

**Authors:** Helen Eyles, Jacqueline Grey, Yannan Jiang, Elaine Umali, Rachael McLean, Lisa Te Morenga, Bruce Neal, Anthony Rodgers, Robert N Doughty, Cliona Ni Mhurchu

**Affiliations:** 1 National Institute for Health Innovation The University of Auckland Auckland New Zealand; 2 Department of Preventive and Social Medicine University of Otago Dunedin New Zealand; 3 Research Centre for Hauora and Health Massey University Wellington Wellington New Zealand; 4 The George Institute for Global Health University of New South Wales Sydney Australia; 5 Department of Medicine The University of Auckland Auckland New Zealand

**Keywords:** mobile health, mHealth, smartphone, smartphone app, cardiovascular disease, sodium, salt, blood pressure, technology, reduced-sodium salt, mobile phone

## Abstract

**Background:**

Even modest reductions in blood pressure (BP) can have an important impact on population-level morbidity and mortality from cardiovascular disease. There are 2 promising approaches: the SaltSwitch smartphone app, which enables users to scan the bar code of a packaged food using their smartphone camera and receive an immediate, interpretive traffic light nutrition label on-screen alongside a list of healthier, lower-salt options in the same food category; and reduced-sodium salts (RSSs), which are an alternative to regular table salt that are lower in sodium and higher in potassium but have a similar mouthfeel, taste, and flavor.

**Objective:**

Our aim was to determine whether a 12-week intervention with a sodium-reduction package comprising the SaltSwitch smartphone app and an RSS could reduce urinary sodium excretion in adults with high BP.

**Methods:**

A 2-arm parallel randomized controlled trial was conducted in New Zealand (target n=326). Following a 2-week baseline period, adults who owned a smartphone and had high BP (≥140/85 mm Hg) were randomized in a 1:1 ratio to the intervention (SaltSwitch smartphone app + RSS) or control (generic heart-healthy eating information from The Heart Foundation of New Zealand). The primary outcome was 24-hour urinary sodium excretion at 12 weeks estimated via spot urine. Secondary outcomes were urinary potassium excretion, BP, sodium content of food purchases, and intervention use and acceptability. Intervention effects were assessed blinded using intention-to-treat analyses with generalized linear regression adjusting for baseline outcome measures, age, and ethnicity.

**Results:**

A total of 168 adults were randomized (n=84, 50% per group) between June 2019 and February 2020. Challenges associated with the COVID-19 pandemic and smartphone technology detrimentally affected recruitment. The adjusted mean difference between groups was 547 (95% CI −331 to 1424) mg for estimated 24-hour urinary sodium excretion, 132 (95% CI −1083 to 1347) mg for urinary potassium excretion, −0.66 (95% CI −3.48 to 2.16) mm Hg for systolic BP, and 73 (95% CI −21 to 168) mg per 100 g for the sodium content of food purchases. Most intervention participants reported using the SaltSwitch app (48/64, 75%) and RSS (60/64, 94%). SaltSwitch was used on 6 shopping occasions, and approximately 1/2 tsp per week of RSS was consumed per household during the intervention.

**Conclusions:**

In this randomized controlled trial of a salt-reduction package, we found no evidence that dietary sodium intake was reduced in adults with high BP. These negative findings may be owing to lower-than-anticipated engagement with the trial intervention package. However, implementation and COVID-19–related challenges meant that the trial was underpowered, and it is possible that a real effect may have been missed.

**Trial Registration:**

Australian New Zealand Clinical Trials Registry ACTRN12619000352101; https://www.anzctr.org.au/Trial/Registration/TrialReview.aspx?id=377044 and Universal Trial U1111-1225-4471

## Introduction

### Background

High blood pressure (BP) is the leading cause of premature and preventable death worldwide [1], mostly owing to its effect on cardiovascular disease (CVD). The relationship between high BP and sodium intake is widely recognized, with long-term reduction of dietary sodium resulting in a decrease in BP regardless of hypertension status, sex, ethnic group, or use of BP-lowering medication [2].

Even modest reductions in BP can have important impacts on population-level morbidity and mortality from CVD [2]. Therefore, in 2013, the World Health Organization (WHO) proposed a target for member states to achieve a 30% relative reduction in population sodium intake toward 2000 mg per day by 2025 [3], and at least 96 countries worldwide are working toward this target through a formal national sodium-reduction strategy [4]. In Aotearoa New Zealand (NZ), an ethnically diverse country of approximately 5 million, adults consume 40% more sodium than WHO recommendations (3373 mg per day) [5,6], and 1 in 5 adults has high BP [7]. Furthermore, high BP and cardiovascular conditions are unequally distributed, with populations traditionally underserved by the health care system, including those from lower-income groups, Māori (indigenous New Zealanders) whānau (families), and Pacific communities, having a higher burden [7]. Although NZ does not have a national sodium-reduction strategy, The Heart Foundation has been working with the food industry for more than a decade to remove sodium from low-cost, high-volume packaged foods [8]. In addition, the Ministry for Primary Industries launched the Health Star Rating front-of-pack nutrition label in 2014 to help consumers make healthier food choices and encourage reformulation [9]. Although some progress has been made from these voluntary initiatives, their impact on total population sodium intake is limited [10,11]. Therefore, effective, scalable, and equitable interventions are urgently needed.

**Figure 1:**
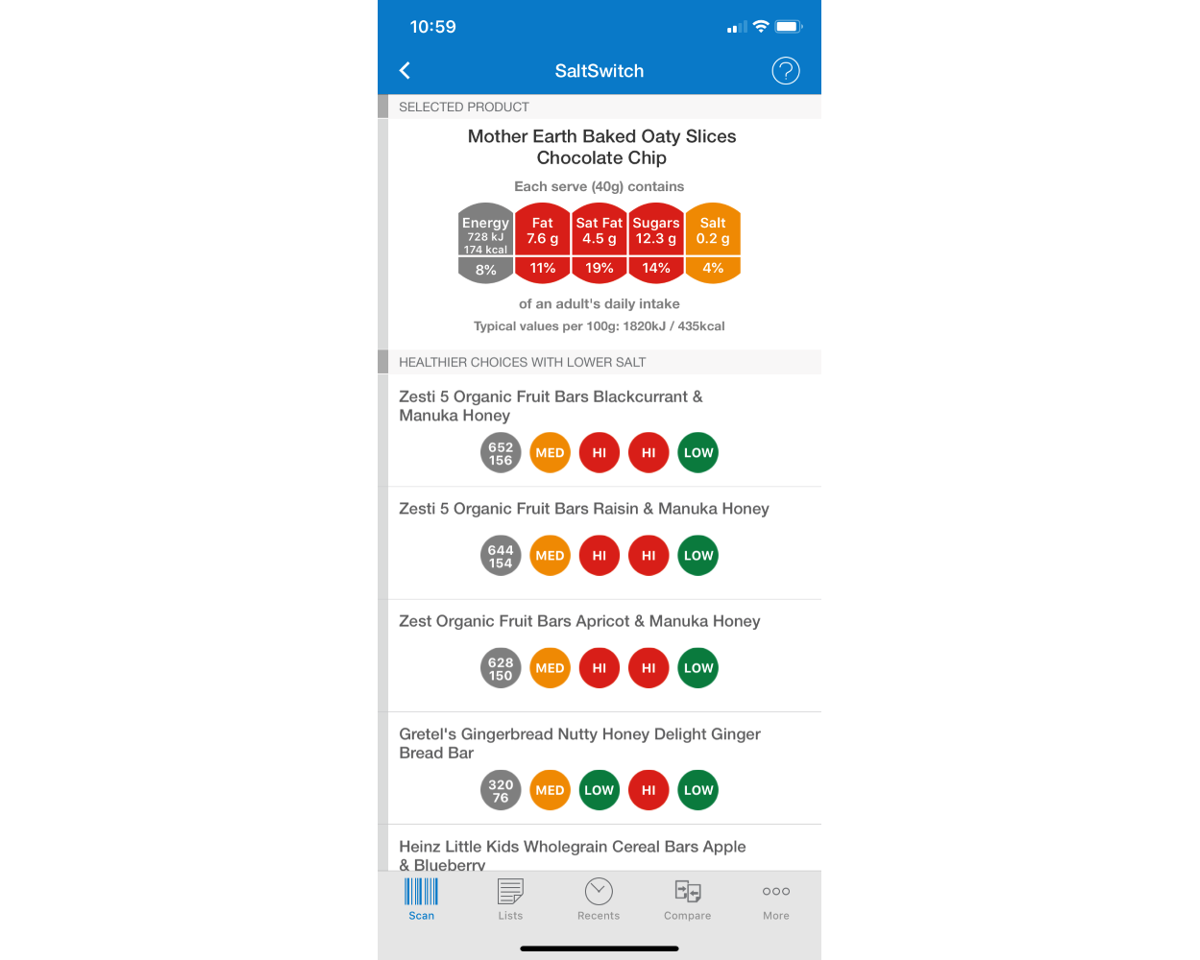
The SaltSwitch smartphone app.

There are 2 promising approaches: mobile health (mHealth) interventions and reduced-sodium salts (RSSs). There is a growing body of evidence suggesting that electronic health and mHealth interventions can support individual changes toward healthier diets [12]. However, there have been few robust randomized controlled trials (RCTs) of app-based interventions, particularly those related to dietary sodium reduction [13,14]. In 2014 and 2015, we conducted a 6-week pilot trial of the effects of the SaltSwitch smartphone app to support adults with diagnosed CVD to make lower-sodium food choices [15]. SaltSwitch (Figure 1) helps consumers choose packaged foods that are lower in sodium; these foods make up approximately 75% of the sodium consumed in NZ, with discretionary salt added during cooking and at the table contributing approximately 15% and the remainder being naturally present in fresh foods [16]. Intervention households in the pilot trial (n=33) purchased significantly less salt from packaged foods (mean 0.3, 95% CI 0.58-0.03 g/MJ) than control households (n=33), supporting a larger trial of the SaltSwitch app with longer-term follow-up (ACTRN12614000206628) [15].

However, SaltSwitch does not address discretionary salt. In contrast, RSSs, or salt substitutes, provide an alternative to regular table salt as some of the sodium chloride has been replaced by potassium salts or other minerals; they are lower in sodium and higher in potassium but have a similar mouthfeel, taste, and flavor. There is evidence from a 2022 Cochrane meta-analysis including 26 RCTs and 34,961 participants showing that the use of an RSS can reduce sodium chloride in the diet by 3% to 77% [16]. A subset of 12 RCTs in the review measured the effects of RSSs on 24-hour urinary sodium and potassium excretion, which ranged from −1730 to +460 mg per day and −170 to +720 mg per day, respectively. A total of 25 RCTs in the review measured BP, with 20 reporting data appropriate for meta-analysis; these studies found that RSSs can reduce systolic BP (SBP) by a mean difference of −4.76 (95% CI −6.01 to −3.5) mm Hg [17]. The wide range of effects was investigated in subgroup analyses, but there was low statistical power, and it was not possible to determine whether some types of RSS interventions were likely to be more effective than others or whether particular populations were most likely to benefit. Furthermore, none of the included studies were from countries where discretionary salt use contributed <25% to dietary sodium intake, such as in NZ.

### Objectives

The primary aim of the Salt Alternatives Study (SALTS) was to determine whether 12 weeks of intervention with a sodium-reduction package (SaltSwitch app + RSS) could reduce estimated 24-hour urinary sodium excretion in adults with high BP (ACTRN12619000352101; Universal Trial U1111-1225-4471).

## Methods

### Study Design

SALTS was a 2-arm parallel RCT conducted in NZ between May 2019 and February 2021. A 2-week baseline period was followed by a 12-week intervention period.

### Ethics Approval

The trial protocol [18] was approved by the NZ Health and Disability Ethics Committees in February 2019 for a period of 3 years (18/NTB/239), and the trial was prospectively registered in the Australian New Zealand Clinical Trials Registry (ACTRN12619000352101).

### Participants and Recruitment

#### Participants

Eligible participants were adults aged ≥18 years who owned a smartphone, had a seated SBP of ≥140 mm Hg or diastolic BP (DBP) of ≥85 mm Hg, planned to undertake household grocery shopping during the trial period, and could read and understand English. The exclusion criteria were SBP of >200 mm Hg; DBP of >120 mm Hg; using an RSS; using the SaltSwitch app; contraindication to altering sodium or potassium intake in the diet; taking furosemide, regular prednisone, or nonsteroidal anti-inflammatory drugs; having had a stroke or cardiovascular event in the previous 6 months; diagnosis of heart failure; planning on being away from home for ≥2 of the subsequent 14 weeks; or inability to provide informed consent. Participants were also excluded at the end of the baseline period if they did not return a spot (casual) urine sample and provide at least 6 home-based BP measures during the baseline period.

#### Recruitment

Participants were recruited from 2 large NZ cities: Auckland and Wellington. Recruitment settings were (1) face to face at community events such as night markets, outside pharmacies, in shopping malls, and via a mobile BP clinic run by the Stroke Foundation of NZ; (2) referrals from general practitioners (GPs) and pharmacists; (3) email invitations sent to staff at the University of Auckland; (4) six Facebook advertising campaigns; (5) two market research panels, Dynata and Horizon; and (6) HealthMatch, a clinical trial participant recruitment company. Specific engagement strategies were adopted to attract participants from Māori whānau and Pacific communities, including attendance at events with Ngāti Whātua Ōrākei (tangata whenua [indigenous people] of Tāmaki Makaurau or Auckland), hauora (well-being) health checks, local markets, and working directly with Pacific health organizations. All participants provided informed consent via the study smartphone app.

### Randomization and Blinding

Eligible participants were randomly assigned in a 1:1 ratio to receive either the sodium-reduction intervention package (SaltSwitch smartphone app + RSS) or the control (generic heart-healthy eating information). Randomization was stratified by ethnicity (Māori and non-Māori) and age (<55 and ≥55 years) using permuted block randomization with variable block sizes of 2 or 4. Participants from Māori whānau and Pacific communities were not grouped for randomization as Pacific communities comprise a smaller proportion of the population and have a lower response rate [19] and Māori are the tangata whenua (original inhabitants) of Aotearoa NZ. The allocation sequence was generated by the study statistician (YJ) using computer-generated randomization lists and concealed in a secure database hosted on REDCap (Research Electronic Data Capture; Vanderbilt University) [20] until the point of randomization. Participants were assigned to trial groups by study research assistants using a REDCap software survey form. As the intervention required dietary change from participants and technology support from the study staff, it was not possible to blind participants or all study staff members to the allocation group. However, the lead study researchers (HE, RM, LTM, BN, AR, RND, and CNM) and trial statistician (YJ) were blinded until trial completion.

### Intervention and Control

#### Intervention

Participants randomized to the intervention received a dietary sodium-reduction package including (1) access to the SaltSwitch smartphone app and (2) a supply of an RSS (as a salt substitute). To encourage the use of SaltSwitch and the RSS, intervention participants were sent weekly reminder notifications to their smartphones. Participants were advised to use the SaltSwitch app whenever they shopped for packaged food brought into the home and to use the RSS in all instances where they would usually use traditional table salt. However, no further dietary advice was provided.

The SaltSwitch app (Figure 1) enables users to scan the bar code of a packaged food using their smartphone camera and receive an immediate, interpretive traffic light nutrition label on-screen alongside a list of healthier, lower-salt options in the same food category. Users can also directly compare the salt content and healthiness of 2 or more foods and create a list of frequently scanned products. SaltSwitch was developed by the George Institute for Global Health [21] and adapted for NZ using the brand-specific Nutritrack (National Institute for Health Innovation) food composition database [22]. Nutritrack is updated annually via cross-sectional surveys of all packaged foods displaying a nutrition information panel sold at the 4 main supermarket chains in NZ (Countdown, New World, PAK’nSAVE, and Four Square) [22]. The Nutritrack database covers approximately 75% of all supermarket food purchases each year. The SaltSwitch food composition data were updated once during the trial. Once downloaded, the SaltSwitch app guided participants through a brief tutorial on how to use the app but did not provide any information on which products to scan. An older, out-of-date version of the SaltSwitch app was available in the NZ Apple and Android app stores during the trial as a component of the NZ FoodSwitch app [21].

The RSS (salt substitute) was manufactured by NuTek Food Science and was a blend of potassium and sea salt, which provided a 75% reduction in sodium compared with regular table salt (74.5% potassium chloride, 24.5% sodium chloride, and 1% silicon dioxide). Intervention participants were sent two 79-g containers of the RSS in plain packaging. The RSS provided to trial participants was not available for commercial sale in NZ during the trial. However, Mrs Rodgers Low Sodium Salt, comprising 49% sodium chloride and 46% potassium and magnesium chloride, was available for sale in some supermarkets.

#### Control

Participants randomized to the control group received a link to generic heart-healthy eating advice developed by the Heart Foundation of NZ sent to control participants’ smartphones during week 1 of the 12-week intervention period. The generic advice was centered on a heart-healthy visual food guide showing the proportion of each type of food to eat each day. The web pages and links also included examples of food types such as grain foods and starchy vegetables, tips on how to achieve a heart-healthy eating pattern, how to read food labels, and how to cut back on salt.

### Study Procedures

#### The Study Smartphone App

**Figure 2:**
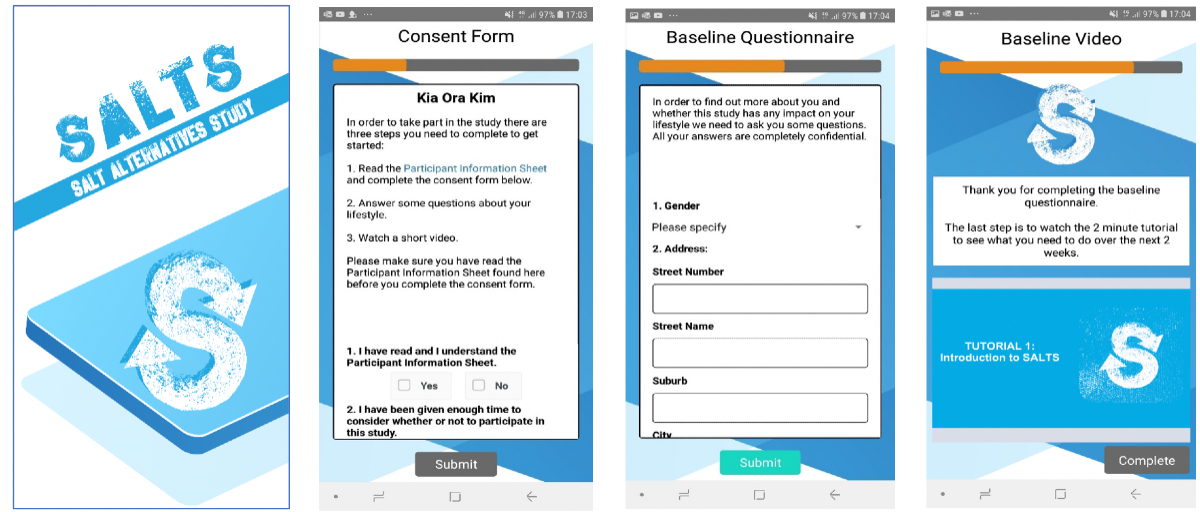
The Salt Alternatives Study (SALTS) smartphone app part one.

**Figure 3:**
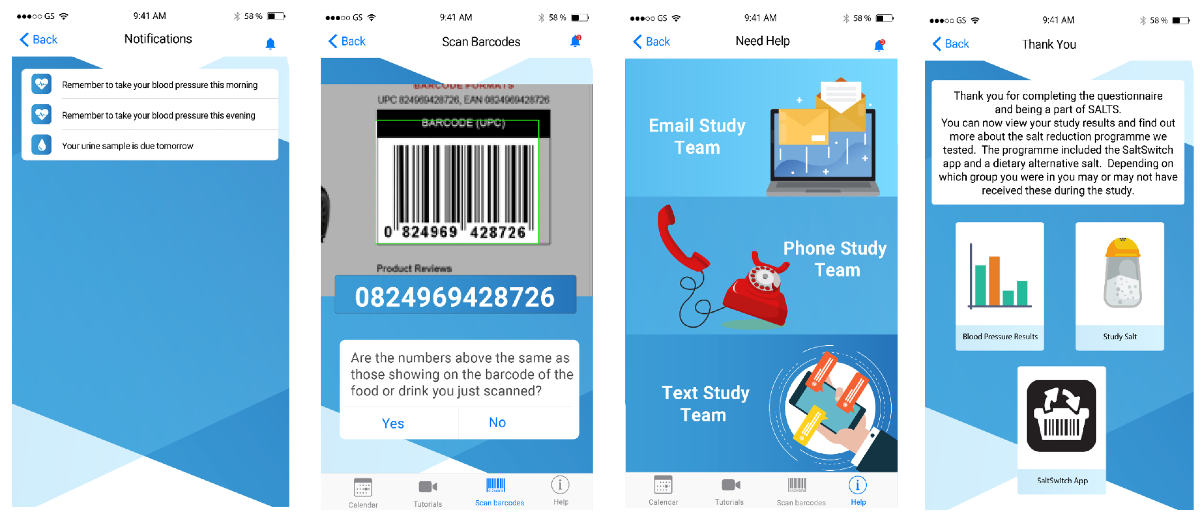
The Salt Alternatives Study (SALTS) smartphone app part two.

A customized study smartphone app was created to assist with the self-return of urine and BP measures and self-completion of questionnaires and support participants with their trial journey (Figures 2 and 3). The following features were included: consent, questionnaires, video tutorials, notification reminders for urine and BP collection, a barcode scanner for packaged foods, study contact information, and (posttrial) information about the intervention package.

#### Referral, Screening, and Consent

Referrals were completed by study research assistants and health care providers using a web form [23] with fields for name, mobile number, email address, smartphone ownership, height, weight, and BP. Height was recorded to the nearest 1 cm, and weight was recorded to the nearest 100 g. Following 5 minutes of rest, the referrers took 3 BP measures on the left arm, and the average of the last 2 was automatically calculated. Individuals were advised to follow up their BP measurements with their GP if their measured SBP was ≥200 mm Hg or DBP was ≥85 mm Hg. Researchers used a standard stadiometer to measure height, a Salter electronic scale to measure weight, and an automated BP monitor [24] to measure BP. The equipment used by health care providers varied. Verbal consent was requested to enable the completed referral forms to be sent to the study researchers.

Early referrals did not attend any trial visits in person. However, from August 2019, referrals were offered a screening visit to assist with the use of the study technology. Screening and enrollment were completed by study research assistants via phone or in person using a web form [23]. Participants who met all screening criteria were sent an SMS text message with a link to download the study smartphone app (Figures 2, 3, and the following sections) and complete consent, after which they were provided with a Blipcare Wi-Fi–enabled BP monitor manufactured by Carematix Inc [24], equipment to collect and return 2 spot urine samples, and instructions for data collection. Phone support was also provided.

#### Baseline

The 2-week baseline period was designed to familiarize participants with trial technologies and collect baseline outcome data. The baseline questionnaire was hosted on REDCap [20] and included date of birth, address, ethnicity, qualifications, employment, household income, behavior regarding dietary salt (excluding total discretionary salt use), existing health conditions, number of household members sharing groceries, concurrent medications, and preferred times for BP measurement reminders. At baseline, participants were asked to scan the bar codes of all packaged foods purchased during the 2-week period, take BP measures in the morning and evening during the second week, and return a spot urine sample from any day during the second week. Potential participants who failed to return all baseline data by 2 weeks after enrollment received a follow-up support phone call; nonresponders 2 weeks after this call were considered lost to follow-up.

#### Follow-up

During the 12-week intervention period, participants were asked to scan the bar codes of all packaged food purchases (weeks 11 and 12), take BP measures in the morning and evening, collect and return a spot urine sample, and complete the follow-up questionnaire (all during week 12). The follow-up questionnaire was hosted on REDCap [20] and included all baseline questions in addition to questions related to the use of meal kits, recent cardiovascular or adverse events, and self-measured body weight. Intervention participants also answered questions about the use and acceptability of the intervention package and the amount of leftover RSS. All participants were provided with a summary of their BP measures, information on where to purchase an RSS, and access to the SaltSwitch smartphone app (removed 3 months after the last participant completed the trial) on trial completion.

### Outcomes

#### Primary Outcome: Estimated 24-Hour Urinary Sodium Excretion

Urinary sodium excretion was measured as a proxy for sodium intake. To reduce participant burden, under- and overcollection, and a low response rate, urinary sodium excretion was estimated via a spot (casual) urine sample rather than measured using a gold-standard 24-hour urine collection [25]. Spot urine samples were collected at any time of day except the first void, chilled by participants, and frozen at −18 °C on receipt. Urine samples were thawed at room temperature, vortexed, and analyzed in batches. Urinary sodium and potassium levels were determined on a Roche Hitachi Cobas C311 unit biochemical analyzer using an ion-selective electrode. Urinary creatinine level was determined through Jaffe reaction using alkaline picrate (Roche Hitachi Cobas C311 analyzer). The concentration of sodium was converted to an estimated 24-hour sodium excretion using a standard urine volume of 1.99 L based on previously reported data for approximately 100 NZ adults [26].

#### Secondary Outcomes

##### Estimated 24-Hour Potassium Excretion

The 24-hour potassium excretion was estimated using the same methods as for the estimated 24-hour sodium excretion.

##### BP: SBP, DBP, and BP Control

BP was measured using a Blipcare Wi-Fi–enabled BP monitor programmed to automatically send readings back to study servers via an application programming interface [24]. Participants collected BP measurements in triplicate 1 minute apart on the left arm after 5 minutes of rest [27] in the morning and evening. Reminder notifications were sent to participants’ smartphones, and if no measures were received, researchers followed up with a phone call and additional notifications. Participants who returned <6 BP measures at baseline were excluded. The definition for BP control (≤135/85 mm Hg) was lower than that used for referral purposes as the latter was taken at home and the former was taken in the community [28].

##### Sodium Content of Packaged Food Purchases

Bar codes for packaged foods purchased for home consumption were collected using a scanning feature in the study smartphone app (Figures 2 and 3). The sodium content of household food purchases was calculated by linking bar codes with Nutritrack [22], the brand-specific NZ food composition database used in the SaltSwitch app (see the *Intervention* section). Weekly reminder notifications were sent to the participants’ smartphones, and if no measures were received, researchers followed up with a phone call and additional notifications.

##### Use and Acceptability of the Intervention Package

Data on the use and acceptability of the SaltSwitch smartphone app and the RSS were collected via the follow-up questionnaire. Bar codes scanned when using the SaltSwitch app were monitored using Google Analytics (for participants who had mobile data available). All intervention participants were asked to record how many teaspoons of RSS they had left at the end of the intervention period.

### Safety and Adverse Events

Participants who reported abnormal BP measures after randomization were telephoned or sent an SMS text message advising them to visit their GP. Abnormal BP measures were defined as (1) consistently elevated SBP (>180 mm Hg for 3 consecutive days, including any missing days), (2) consistently low SBP (<90 mm Hg for 3 consecutive days, including any missing days), or (3) major changes in SBP from baseline (>20 mm Hg). The salt-reduction package was considered low risk. Therefore, only serious adverse events were collected via the follow-up questionnaire and reported to the Ethics Committee annually. A qualified medical representative was authorized to determine whether adverse events were considered serious.

### Statistical Analysis

#### Sample Size

A total of 326 participants (163 per group) were estimated to provide 80% power at a 5% level of significance (2-sided) to detect a minimum effect size of 462 mg of sodium in the primary outcome between the 2 groups, allowing for a 10% loss to follow-up. The expected effect size was estimated from the SaltSwitch pilot study data, where estimations of 24-hour urinary sodium excretion were calculated using spot urine samples and a standard urine volume of 1.99 L with an SD of 1400 mg per day (ACTRN12614000206628) [15].

#### Main Comparative Analyses

All participant data collected at baseline and week 12 were summarized using descriptive statistics for the intervention and control groups separately. Continuous variables were presented as mean and SD, whereas categorical variables were reported as frequencies and percentages.

The trial evaluation was performed on an intention-to-treat basis, including all eligible participants in the group to which they were randomized. Multiple imputation methods were used for missing primary outcome data in the primary intention-to-treat analysis using the Markov chain Monte Carlo method and assuming that the data were missing at random. No imputation was considered on secondary outcomes. Sensitivity analysis was conducted on the primary outcome (1) without imputation and (2) using the International Cooperative Study on Salt and Blood Pressure formula [29] rather than a standard volume to estimate 24-hour sodium excretion. Linear regression was used for continuous outcomes adjusting for baseline outcome value, age, and ethnicity (stratification factors). The model-adjusted mean difference between the 2 groups was estimated with a 95% CI and *P* value. Logistic regression was used for categorical outcomes, and the estimated group difference was reported as the odds ratio. Owing to the small sample size, no subgroup analysis was considered.

The definition of valid data for spot (casual) urine samples was a collection at baseline during week −1 (−2 weeks to +2 weeks) and at follow-up during week 12 (−1 week to +1 week). Valid BP measurements at baseline were those taken during weeks −2 and −1 (−2 weeks to +2 weeks) and at follow-up during week 12 (−1 week to +1 week). The average SBP and DBP were calculated using a minimum of 6 readings at each time point. Valid bar code data to estimate the sodium content of household food purchases at baseline were scanned during week −1 (−2 weeks to +2 weeks) and at follow-up during week 12 (−1 week to +1 week). The average sodium content of food purchases was calculated for all bar codes received.

Statistical analyses were performed using SAS (version 9.4; SAS Institute). All statistical tests were 2-sided at a 5% significance level.

### Changes in Response to the Challenges of the COVID-19 Pandemic

As recommended by Perlis et al [30], we outline the challenges associated with the COVID-19 pandemic and how these affected the SALTS trial. In NZ, there were strict lockdown periods from March 2020 to June 2020, from August 2020 to October 2020, in February 2021, and from August 2021 to November 2021. During these times, most postreferral and data collection procedures could be completed using remote technology. However, lockdown periods prevented enrolled participants from returning spot urine samples as couriers were only available for essential activities, and the university campus was closed, meaning that samples could not be received. Lockdown periods also substantially compromised recruitment as they prevented the collection of face-to-face BP measures necessary for new referrals. Consequently, recruitment was put on hold during these times. Furthermore, potential participants who had been referred and identified as eligible were unable to start the trial during lockdowns as it was not possible for researchers to courier the Wi-Fi–enabled BP monitor and equipment to collect urine samples; as a result, a considerable number of eligible participants lost interest and declined to take part, and recruitment was further compromised (see the *Recruitment* section).

## Results

Trial results are reported according to the CONSORT (Consolidated Standards of Reporting Trials) 2010 guidelines for parallel-group randomized trials [31].

### Recruitment

**Figure 4:**
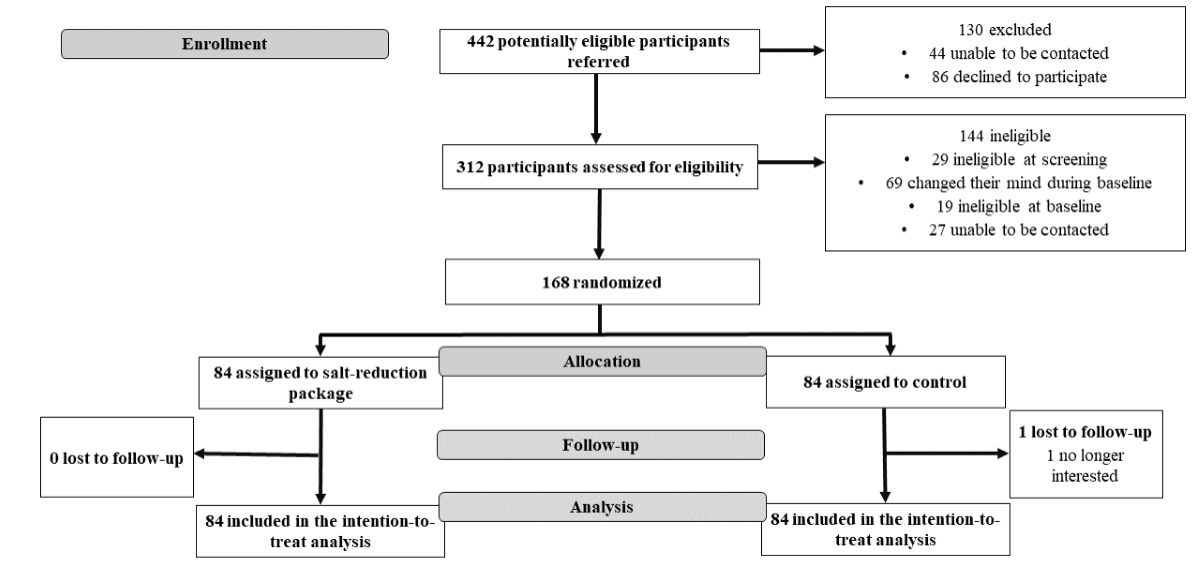
Trial profile.

Recruitment took place over 16 months, starting on May 30, 2019, and finishing on October 2, 2019. The last participant was randomized in February 2020. A total of 442 potentially eligible participants were referred, of whom 312 (70.6%) were screened for initial eligibility, 86 (19.5%) declined to participate, and 44 (10%) were unable to be contacted (Figure 4). Of the 312 screened participants, 144 (46.2%) were ineligible as they changed their mind during baseline (n=69, 22.1%), did not meet the screening criteria (n=29, 9.3%), were unable to be contacted (n=27, 8.7%), or did not provide the required baseline data (n=19, 6.1%). The remaining 53.8% (168/312) of the initially screened eligible participants were randomized and took part in the trial; of those, the largest number was from market research panels (55/168, 32.7%) followed by face-to-face events (24/168, 14.3%). The final participant completed the trial on March 20, 2021 [31,32].

### Baseline Characteristics of Trial Participants

A total of 84 (50%) of the 168 participants were randomized to the intervention group, and the remaining 84 (50%) were randomized to the control group. A participant in the control group withdrew, stating that they were no longer interested in taking part. A total of 14.3% (24/168) of the participants identified as Māori, and 7.1% (12/168) identified as Pacific (7/84, 8% in the control group and 5/84, 6% in the intervention group). All participant characteristics were similar between groups (Table 1).

**Table 1:** Baseline characteristics of the trial participants (N=168).

				Control group (n=84)		Intervention group (n=84)	
**Baseline characteristics**							
	**Age (years), mean (SD)**			55 (13)		54 (13)	
		18 to 54, n (%)		38 (45)		37 (44)	
		≥55, n (%)		46 (55)		47 (56)	
	**Gender, n (%)**						
		Men		41 (49)		36 (43)	
		Women		36 (43)		38 (45)	
		Nonbinary or not specified		7 (8)		10 (12)	
	**Region, n (%)**						
		Auckland		71 (85)		76 (90)	
		Other New Zealand		13 (15)		8 (10)	
	**Smartphone ownership, n (%)**						
		iPhone		32 (38)		41 (49)	
		Android		52 (62)		43 (51)	
	**Prioritized ethnicity^a^, n (%)**						
		Māori		12 (14)		12 (14)	
		Pacific		7 (8)		5 (6)	
		Asian		14 (17)		15 (18)	
		European or other		51 (61)		52 (62)	
	**Highest qualification, n (%)**						
		None		5 (6)		1 (1)	
		Secondary		11 (13)		10 (12)	
		University degree, polytechnic, trade, or diploma		44 (52)		43 (51)	
		Postgraduate degree		21 (25)		27 (32)	
		Other		3 (4)		3 (4)	
	**Employment status, n (%)**						
		Full- or part-time employment		57 (68)		58 (69)	
		Retired or full-time homemaker		17 (20)		14 (17)	
		Unemployed or student		9 (11)		12 (14)	
		Decline to answer		1 (1)		0 (0)	
	**Annual household income (NZ $ [US $]), n (%)**						
		≤60,000 (US $37,826.7)		20 (24)		18 (21)	
		60,001 to 100,000 (US $37,827.33 to US $63,036.80)		21 (25)		21 (25)	
		≥100,001 (≥US $63,037.43)		30 (36)		34 (40)	
		Declined to answer		13 (15)		11 (13)	
	**Behaviors regarding dietary salt, n (%)**						
		**Add salt to food**					
			Always or often		35 (42)		35 (42)
			Sometimes		23 (27)		19 (23)
			Rarely or never		26 (31)		30 (36)
		**Salt added during cooking**					
			Always or often		52 (62)		54 (64)
			Sometimes		23 (27)		16 (19)
			Rarely or never		9 (11)		14 (17)
		**Saltshaker placed on table**					
			Always or often		27 (32)		28 (33)
			Sometimes		16 (19)		14 (17)
			Rarely or never		40 (48)		42 (50)
			Do not know		1 (1)		0 (0)
		**Trying to cut down the amount of salt consumed**					
			No		39 (46)		34 (40)
			Yes		40 (48)		36 (43)
			Do not know		5 (6)		14 (17)
		**Look at nutrition information on food packages**					
			Always or more often than not		29 (35)		25 (30)
			Occasionally		38 (45)		50 (60)
			Never		17 (20)		9 (11)
**Baseline clinical measures**							
	Height (cm), mean (SD)			169 (10)		168 (9)	
	Weight (kg), mean (SD)			88 (19)		89 (18)	
	BMI (kg/m^2^), mean (SD)			31 (6)		31 (5)	
	**Blood pressure (mm Hg)^b^, mean (SD)**						
		Systolic		142 (13)		143 (16)	
		Diastolic		86 (9)		86 (9)	
	**Estimated 24-hour urine excretion (mg per day)^c^, mean (SD)**						
		Sodium		3107 (1917)		3616 (2280)	
		Potassium		3601 (2202)		4232 (2659)	
	**Current health condition^d^, n (%)**						
		Diabetes		12 (14)		10 (12)	
		High cholesterol		26 (31)		29 (35)	
		High blood pressure		54 (64)		60 (71)	

^a^Participants were allocated to a single ethnic group in the following order of priority even if they identified with more than one ethnicity: Māori, Pacific, Asian, and European or other.

^b^Valid blood pressure data only (ie, received within 1 week before and 2 weeks after randomization date and including a minimum of 6 readings).

^c^Valid urine data only (ie, received within 1 week before and 2 weeks after randomization date); n=73 for the control group and n=77 for the intervention group. Estimated from the concentration of 1 spot urine sample and a standard volume of 1.99 L, with no adjustment for electrolytes not excreted via urine.

^d^As advised by a health professional.

### Return of Trial Outcome Data

Valid urine samples for the estimation of sodium and potassium excretion were returned by 89.3% (150/168) of the participants at baseline and 45.2% (76/168) of the participants at follow-up. More participants in the control group compared with the intervention group returned a valid urine sample at baseline (77/84, 92% vs 73/84, 87%, respectively; Table 2). Valid BP data were returned by 93.5% (157/168) of the participants at baseline and 83.3% (140/168) of the participants at follow-up. Valid bar code data for the estimation of the sodium content of household food purchases were returned by 76.2% (128/168) of the participants at baseline and 22% (37/168) of the participants at follow-up. The baseline questionnaire was completed by 100% (168/168) of the participants, and the follow-up questionnaire was completed by 76.2% (128/168) of the participants. The rate of return of follow-up data was consistent across ethnic groups except for the follow-up questionnaire, which was returned by 67% (24/36) of the participants identifying as Māori or Pacific and 78.8% (104/132) of the participants identifying as all other ethnicities.

**Table 2:** Estimates of the effect of the salt-reduction intervention package on urinary sodium and potassium excretion, blood pressure, and the sodium content of household packaged food purchases at 12 weeks (N=168).

			Control group (n=84)					Intervention group (n=84)					Adjusted difference at 12 weeks^a^ (95% CI)
			Baseline		12 weeks		Baseline			12 weeks			
			Participants with valid data, n (%)^b^	Mean (SD)	Participants with valid data, n (%)^b^	Mean (SD)	Participants with valid data, n (%)^b^		Mean (SD)	Participants with valid data, n (%)^b^	Mean (SD)		
**Primary outcome: estimated 24-hour sodium excretion (mg per day)**													
	**Standard volume**		73 (87)	3107 (1917)	38 (45)	3193 (2284)	77 (92)		3616 (2280)	38 (45)	3935 (2268)		
		Multiple imputations (primary)^c^										547 (−331 to 1424)	
		No imputation^d^										670 (−304 to 1645)	
	**INTERSALT^e^ formula**		65 (77)	3324 (813)	35 (42)	3222 (857)	66 (79)		3320 (782)	32 (38)	3289 (792)		
		No imputation^f^										24 (−254 to 302)	
**Secondary outcomes**													
	Estimated 24-hour potassium excretion (mg per day)		73 (87)	3601 (2202)	38 (45)	4078 (2945)	77 (92)		4232 (2659)	38 (45)	4210 (2334)	132 (−1083 to 1347)	
	Systolic blood pressure (mm Hg)^g^		78 (93)	142 (13)	60 (71)	138 (15)	79 (94)		143 (15)	60 (71)	139 (12)	−0.66 (−3.48 to 2.16)	
	Diastolic blood pressure (mm Hg)^g^		78 (93)	86 (9)	60 (71)	84 (10)	79 (94)		86 (9)	60 (71)	84 (8)	−0.35 (−2.20 to 1.50)	
	Sodium content of household food purchases (mg per 100 g)^h^		67 (80)	346 (427)	20 (24)	253 (326)	61 (73)		350 (536)	17 (20)	262 (151)	73 (−21 to 168)	

^a^Linear regression models adjusted for baseline outcome, age in years, and Māori or Pacific ethnicity.

^b^Valid urine, blood pressure (BP), and food purchasing data were collected within 1 week before or 2 weeks after randomization (for baseline) and 1 week before or 2 weeks after week 12 (for follow-up). For BP, a minimum of 6 readings during these time frames was considered valid. For food purchases, a minimum of 10 products scanned during these time frames was considered valid.

^c^Estimated 24-hour sodium excretion from spot urine using a standard volume of 1.99 L. Multiple imputations used on missing primary outcome data through an intention-to-treat analysis using the Markov chain Monte Carlo method and assuming data were missing at random.

^d^24-hour sodium excretion from spot urine using a standard volume of 1.99 L. No imputation for missing data.

^e^INTERSALT: International Cooperative Study on Salt and Blood Pressure.

^f^24-hour sodium excretion estimated using the INTERSALT formula. No imputation for missing data.

^g^BP control was defined as <135/85 mm Hg. The mean number of valid days for all BP measures at baseline was 12 (SD 6) in the control group and 13 (SD 7) in the intervention group. The corresponding values at week 12 were 8 (SD 3) and 9 (SD 5), respectively. The mean number of valid readings for all BP measures at baseline was 31 (SD 21) in the control group and 37 (SD 24) in the intervention group. The corresponding values at week 12 were 19 (SD 12) and 24 (SD 16), respectively.

^h^The mean number of food products scanned at baseline was 27 (SD 18) in the control group and 24 (SD 18) in the intervention group. The corresponding values at week 12 were 18 (SD 16) and 13 (SD 12), respectively.

### Primary Outcome: Estimated 24-Hour Urinary Sodium Excretion

The mean estimated 24-hour urinary sodium excretion at 12 weeks was 3935 (SD 2268) mg per day in the intervention group and 3193 (SD 2284) mg per day in the control group (Table 2). There was no significant difference between the groups in estimated 24-hour sodium excretion at 12 weeks (adjusted mean difference=547 mg per day, 95% CI −331 to 1424; Table 2).

Sensitivity analyses were consistent with the primary analysis, with no significant differences observed in the mean difference between groups where no imputation was used or where 24-hour urinary sodium excretion was estimated using the International Cooperative Study on Salt and Blood Pressure formula [29] rather than a standard volume of 1.99 L (Table 2).

### Secondary Outcomes

#### Estimated 24-Hour Urinary Potassium Excretion

The mean estimated 24-hour urinary potassium excretion at 12 weeks was 4210 (SD 2334) mg per day in the intervention group and 4078 (SD 2945) mg per day in the control group (Table 2). There was no significant difference between the groups in estimated 24-hour potassium excretion at 12 weeks (adjusted mean difference=132 mg per day, 95% CI −1083 to 1347; Table 2).

#### BP: SBP, DBP, and BP Control

The mean SBP for the intervention and control groups at 12 weeks was 139 (SD 12) mm Hg and 138 (SD 15) mm Hg, respectively (Table 2). The corresponding figures for DBP were 84 (SD 8) mm Hg and 84 (SD 10) mm Hg, respectively. No significant difference was observed between the groups for SBP or DBP at 12 weeks. The adjusted mean difference between groups was −0.7 (95% CI −3.5 to 2.2) mm Hg for SBP and −0.4 (95% CI −2.2 to 1.5) mm Hg for DBP (Table 2). The mean number of participants achieving BP control at 12 weeks in the intervention and control groups was 23 (SD 27) and 17 (SD 20), respectively. There was no significant difference between the groups in the odds of achieving BP control (adjusted odds ratio 1.0, 95% CI 0.45-2.1).

#### Sodium Content of Packaged Food Purchases

The mean number of all bar codes scanned at baseline was 24 (SD 18) for the intervention group and 27 (SD 18) for the control group. The corresponding mean values for the follow-up period were 13 (SD 12) and 18 (SD 16), respectively. There was no significant difference between the groups in the sodium content of packaged foods purchased (adjusted mean difference=73, 95% CI −21 to 168 mg per 100 g; Table 2).

#### Use and Acceptability of the Intervention Package

A total of 76% (64/84) of the intervention participants provided use and acceptability data; of these 64 participants, 48 (75%) reported using SaltSwitch when shopping, with 25 (52%) of them reporting that they used the app “at least half to every time” they shopped (Table 3). Google Analytics data were available for 96% (46/48) of the SaltSwitch users, who scanned a mean of 29 (SD 40) products during the 12-week intervention period over a mean of 6 (SD 6) shopping occasions. The most common responses from the 56% (27/48) of participants who reported what they “liked most” or “least” about SaltSwitch were that it helped with making lower-salt food choices and thinking about salt in food in general (5/27, 19%) but needed more products to be available in the app to scan (10/27, 37%).

A total of 94% (60/64) of the intervention participants who provided data used the RSS, with 69% (44/64) stating that between half and all the discretionary salt they consumed during the 12-week study period was the RSS. Of those who reported using less than half or none of the RSS (20/64, 31%), 20% (4/20) stated that this was because the taste was unacceptable (Table 3). Participants used a mean of approximately 37.2 g (6.5 tsp) of RSS over the 12-week intervention period, and 44% (28/64) stated that their study salt was consumed by other household members.

**Table 3:** Use and acceptability of the salt-reduction intervention package (n=64^a^).

				Values
**SaltSwitch smartphone app, n (%)**				
	Used the SaltSwitch app when grocery shopping over the past 12 weeks (n=64)		48 (75)	
	**How often used? (n=48)**			
		More than half to every time	15 (31)	
		Half of the time	10 (21)	
		A handful of times to less than half of the time	22 (46)	
		Did not answer	1 (2)	
	**How easy to use? (n=48)**			
		Very easy to somewhat easy	33 (69)	
		Neither easy nor difficult	9 (19)	
		Somewhat difficult to very difficult	5 (10)	
		Did not answer	1 (2)	
	Think SaltSwitch is a good way to help shoppers make lower-salt food choices (n=64)		52 (81)	
**Reduced-sodium salt (study salt)**				
	**Amount of salt consumed over the past 12 weeks that was study salt (n=64), n (%)**			
		All or nearly all	35 (55)	
		Half	9 (14)	
		Less than half	16 (25)	
		None	4 (6)	
	**If less than half or none, what was the main reason for this? (n=20), n (%)**			
		Taste unacceptable	4 (20)	
		Unwilling or other reason	16 (80)	
	Teaspoons of salt left at end of study (n=56), mean (SD)^b^		19.8 (19.9)	
	Other household members used the study salt (n=64), n (%)		28 (44)	
	**How many household members used the study salt? (n=28), n (%)**			
		1 to 2	17 (61)	
		3	4 (14)	
		≥4	7 (25)	

^a^A total of 64 intervention participants returned the follow-up questionnaire.

^b^Intervention participants were provided with 158 g or approximately 26.3 tsp of salt.

### Effects for Māori and Pacific Participants

Owing to the low engagement and recruitment of participants from Māori whānau (28/168, 16.7%) and Pacific communities (13/168, 7.7%), it was not possible to estimate differences in effects separately for these groups.

### Adverse Events

No serious adverse events were reported during the trial period.

### Challenges Associated With the Use of Trial Technology

Technology was used in the SALTS trial to streamline study processes, deliver the intervention, collect outcome data, and communicate with participants. Information on the challenges encountered owing to the use of technology, which affected all 4 stages of the SALTS trial and CONSORT flow diagram, [31] is summarized in Table 4 and has been reported elsewhere [32]. However, briefly, during enrollment and allocation of participants (stages 1 and 2), many participants lacked confidence in their ability to download the study smartphone app and connect the Wi-Fi–enabled BP monitor. During this stage, inefficiencies were also experienced by the researchers as the study data management system could not directly exchange information with the referral form and participant tracking systems. During stage 3 (follow-up and collection of outcome data), the study app performed inconsistently across different smartphone models and operating systems, and some participants did not switch on their phone notifications, meaning that they missed important study reminders. Finally, during stage 4 (data analysis), some participants did not complete the follow-up questionnaire or return BP measures within the time frames for valid data as prespecified in the study protocol [31].

**Table 4:** Challenges associated with the use of technology and future recommendations.

Trial stage	Technology challenge	Future recommendations
Enrollment and allocation of participants	Not all smartphone owners use smartphone apps, and use may be lower in older populations. Face-to-face support may be required for confident connection and use of technologies such as smartphone apps and other Wi-Fi–enabled devices. Interoperability, or the exchange of information between technologies, is critical to harness the efficiencies they offer.	Complete background research on the population of interest to understand their use of smartphone technology before using it widely in a research study. Plan for flexibility in the study design to enable face-to-face support for familiarization with study technology, particularly during the early phases. Incorporate funds and time in the study setup phase to ensure that technologies that need to exchange information with one another can do so correctly and efficiently. For example, ensure that web-based forms exchange data with data management systems and data management systems exchange data with participant booking systems. If funds and time cannot be included, consider the use of simple existing tools such as survey software and an ad hoc SMS text messaging service.
Following up participants and collecting outcome data	Technology can behave in unanticipated ways in response to the variety of smartphone models and operating systems on the market, and it can be difficult to replicate “live” trial conditions for all individual circumstances. Not all smartphone users like or read notifications.	Create technology test plans and implement them during all phases of the trial, from early development to the completion of the last participant. When testing technology, use Apple and Android phones and include different operating systems. Have a “soft” launch to enable rigorous early testing with a small group of real participants. To avoid the impacts of software fixes on unrelated functionality, build technology in separate blocks of code that only connect where necessary. Where possible, use SMS text messages rather than notifications to convey key study information to participants, particularly for those with limited Wi-Fi or data.
Data analysis	The flexibility that technology provides to return outcome data at the participants’ convenience can increase the time frame for data return and the variability in measures.	Set realistic time frames or windows for participants to return remote data to researchers. For example, for participants returning a casual urine sample by courier, a realistic number of days will be needed to provide the participant with options, they may need a reminder messages, or there could be courier delays. Set time frames for each outcome that is collected remotely and specify these before study start in the statistical analysis plan. In addition to using standardized methods for the collection of clinical outcome data, consider whether other aspects of outcome data collection should also be standardized. For example, blood pressure measures vary considerably between and within individuals and from day to day and even hour to hour; in this case, standardizing the time for data collection (eg, 8 AM), rather than allowing participants to choose a time in the morning that suits them, will result in reduced variation in blood pressure measures across the sample.

## Discussion

### Principal Findings

In this RCT, we found no evidence that 12 weeks of intervention with a salt-reduction package reduced estimated 24-hour urinary sodium excretion in adults with high BP. The estimated mean sodium excretion was higher in the intervention group than in the control group at 12 weeks; however, the CI for the mean difference was wide, suggesting no real difference. In addition, we found no effect of the intervention package on any secondary outcome, including estimated 24-hour urinary potassium excretion and BP. Although most intervention participants reported using the SaltSwitch app (48/64, 75%) and the RSS (60/64, 94%) during the 12-week intervention period and acceptability of the intervention was high, the intervention dose was low; participants reported using SaltSwitch on less than half of shopping occasions and consuming only approximately 1/2 tsp of RSS per household per week during the intervention period. The low recruitment of participants from Māori whānau and Pacific communities meant that it was not possible to estimate differences in effects separately for these groups.

### Limitations

In addition to the low intervention dose, important limitations of the SALTS trial were the reduced study power, low number of participants from Māori whānau and Pacific communities, lower-than-anticipated engagement with trial technologies, and use of a spot rather than 24-hour urine sample for estimation of the primary outcome. Implementation and COVID-19 challenges meant that the trial was substantially underpowered, and thus, it is possible that a real effect may have been missed. Although a larger trial would have enabled the study hypothesis to be tested as intended, it is possible that the null trial findings would have been similar because of the limited use of intervention components. Given the high acceptability, the reason for the low consumption of the RSS by trial participants is not clear, but the low use of discretionary salt at baseline by trial participants may have been a factor. The ease of use and acceptability of SaltSwitch were also high, but reported use was low, with the reasons most reported by participants for not using the intervention app more often being the use of web-based grocery shopping instead, others completing the grocery shopping, difficulty in downloading or using SaltSwitch, and lack of time.

Despite adopting specific recruitment strategies to attract and engage Māori whānau and Pacific communities, we randomized only 14.3% (24/168) and 7.1% (12/168) of the total participants from these groups, respectively. Although we were able to attend numerous face-to-face events with Māori communities and work directly with Pacific health organizations, a capacity-building approach led by Māori for Māori and by Pacific for Pacific where these groups take an active part in the research would likely have been more effective [33].

Challenges associated with the considerable use of technology in the trial also affected recruitment, engagement, and return of outcome data. However, it is difficult to know the magnitude of the effect related to these challenges as technology provides certain inherent efficiencies and enabled researchers to continue some aspects of the trial during COVID-19 lockdowns.

The use of spot urine rather than gold-standard 24-hour urine samples may have affected the ability to identify a difference in sodium intake between the intervention and control groups [34]. Although 24-hour urine samples are considered too burdensome for nonclinical study populations, future similar research would benefit from a subsample of participants providing a 24-hour urine sample, which would provide some information on the consistency of effects.

Caution should also be exercised when generalizing the findings of the SALTS trial to other population groups. In addition to the low number of participants from Māori whānau and Pacific communities, the study sample was highly educated; many were already trying to cut down on the amount of salt they consumed and using nutrition information on food packages to make healthier choices. Furthermore, 1 in 3 referrals declined to take part or were unable to be contacted, and the inclusion-to-randomization rate (calculated from the 59 referrals who were initially eligible and completed the baseline questionnaire but were not randomized) was lowest for those from Māori whānau (24/52, 46%) and Pacific communities (12/17, 71%; conversion rates for Asian and European or other were 29/36, 81% and 103/132, 78%, respectively). Possible reasons for why almost half of Māori referrals (24/52, 46%) and approximately 40% of Pacific referrals (12/17, 71%) did not continue in the study include the collective cultures of these groups misaligning with the individual framing of the intervention and the lack of face-to-face contact [32]. Although smartphone apps offer benefits, including lower scale-up costs, personalized health information, real-time delivery of advice, and remote assessment of outcomes, there is evidence indicating that face-to-face relationships are a key component for achieving social connection and digital inclusion, both of which are vital to reducing inequities [35,36].

### Strengths

Nonetheless, the SALTS trial is one of the few RCTs of a smartphone app to promote dietary sodium reduction in adults [13]. To date, most mHealth interventions aimed at reducing sodium consumption have focused on improving knowledge and awareness of dietary salt intake using SMS text messaging. The use of more innovative technologies and rigorously designed trials were identified as key recommendations for future research in a 2019 systematic review [14]. Furthermore, SALTS is one of a limited number of trials testing an RSS in a country with a predominantly Western diet where packaged foods contribute most (>50%) of sodium to dietary intake [37].

### Comparison With Prior Work

The null findings of the SALTS trial are inconsistent with those of 2 recent systematic reviews of mHealth interventions that specifically target dietary salt reduction. The first was a 2019 systematic review of the effectiveness of mHealth technologies for salt reduction and included 6 RCTs and 5 quasi-experimental studies; 8 of 11 studies produced positive results [14], and 2 of the RCTs stated salt consumption as the primary outcome (one of which was a pilot of the SaltSwitch app [15]), finding significant reductions in intake as estimated by a spot (casual) urine sample. However, both trials were small (ie, <100 participants). The results of a more recent (2020) RCT of the “LowSalt4Life” just-in-time adaptive mobile app for adults with hypertension were also positive, with a significant reduction in estimated 24-hour urinary sodium excretion compared with usual dietary advice [38]. A 2019 systematic review examining the effectiveness of electronic health interventions for BP control also found a significant overall reduction in sodium intake (n=15 trials) [13].

The reasons why the SaltSwitch app was ineffective compared with previous studies are difficult to determine as the intervention components associated with effectiveness were not investigated. However, in contrast to the SALTS intervention, most previous studies were co-designed or included at least one of the following behavior change techniques: system-generated feedback based on current behaviors, goal setting, regular motivational SMS text messages, or face-to-face support from a health care provider in addition to interaction through an electronic device [13]. A recent (2020) systematic review of mHealth RCTs supports the inclusion of behavior change techniques, with prompts or cues, general personalization, goal setting, and action planning found to be significantly associated with positive change [39]. The SaltSwitch app included only one of these techniques (prompts or cues to use the app), which may help explain its lack of efficacy. The feasibility of an app called SaltSwap combined with a brief behavioral intervention was recently explored in adults with high BP in the United Kingdom. Although researchers found no evidence that the intervention reduced dietary salt intake, findings of a future adequately powered trial will provide further information on the effectiveness of mHealth interventions based on behavioral theory [40]. The contrasting positive findings of the SALTS pilot trial also suggest that individual-level behavior change interventions may be more beneficial for highly motivated clinical populations [15,41]. Approaches likely to be more successful at the population level include those outlined by the WHO in their Surveillance, Harness Industry, Adopt Standards for Labelling and Marketing, Knowledge, Environment technical package for salt reduction (ie, regular measuring and monitoring of population salt use, reformulation of foods and meals to contain less salt, effective food labeling and marketing, education campaigns, and supporting settings such as hospitals and universities to promote healthy eating [42]).

The null findings of the SALTS trial are also inconsistent with evidence from a 2022 Cochrane meta-analysis, which showed that the use of an RSS can reduce urinary sodium excretion by up to 1730 mg per day [17]. However, none of the included studies were from countries such as NZ, where discretionary salt use contributes <25% to dietary sodium intake, and there were insufficient data in the review to determine whether the type of RSS or study population affected effectiveness. The limited use of the RSS by participants in the SALTS trial suggests that, in Aotearoa NZ, it may be more efficacious to focus on the use of RSSs in packaged foods rather than or as an adjunct to a replacement for traditional table salt. That said, RSSs may still be helpful for specific communities or in settings where most food is cooked or prepared in the home or on-site. In settings where RSSs are found to be effective, political actions to support implementation include understanding the path to market and removing cost and accessibility barriers for consumers and food companies [43].

### Conclusions

In summary, our trial found no evidence of the effectiveness of a salt-reduction intervention package comprising a smartphone app and RSS on estimated 24-hour sodium excretion (or any secondary outcome assessed) in adults with high BP. The trial was underpowered because of challenges associated with the implementation of trial technologies and the COVID-19 pandemic, meaning that it is possible that a real effect may have been missed. Furthermore, because of low engagement and recruitment, it was not possible to determine potential effects for Māori whānau or Pacific communities. However, it is also possible that a larger trial in the same study population would produce similar results given the low intervention dose. Nonetheless, further research may be warranted to explore the efficacy of SaltSwitch for secondary prevention in highly motivated clinical populations such as those who have had a recent cardiac event. Further research should also be undertaken to explore the use of RSSs in packaged foods, especially for countries such as NZ, where these foods contribute >50% to population sodium intake, and in specific communities or in settings where most food is cooked in the home or prepared on-site. Finally, the challenges associated with the design and delivery of effective individual-level behavioral interventions highlight the need for comprehensive policies and programs, including improvements to food environments and systems, in addition to supportive tools for behavior change—this is critical if we are to achieve meaningful reductions in population sodium intake.

## Appendix

Multimedia Appendix 1 CONSORT-EHEALTH (Consolidated Standards of Reporting Trials of Electronic and Mobile Health Applications and Online Telehealth) questionnaire V1.6.1.

## Abbreviations

BP: blood pressure
CONSORT: Consolidated Standards of Reporting Trials
CVD: cardiovascular disease
DBP: diastolic blood pressure
GP: general practitioner
mHealth: mobile health
NZ: New Zealand
RCT: randomized controlled trial
REDCap: Research Electronic Data Capture
RSS: reduced-sodium salt
SALTS: Salt Alternatives Study
SBP: systolic blood pressure
WHO: World Health Organization
